# Exploring Key Factors for Contractors in Opening Prefabrication Factories: A Chinese Case Study

**DOI:** 10.3389/fpubh.2022.837350

**Published:** 2022-02-03

**Authors:** Jiasheng Zhang, Pengcheng Xiang, Jia Zhong, Jian Zhang, Zezhou Wu, Maxwell Fordjour Antwi-Afari

**Affiliations:** ^1^School of Management Science and Real Estate, Chongqing University, Chongqing, China; ^2^China Construction Fourth Engineering Bureau No. 3 Co. Ltd., Zunyi, China; ^3^China State Construction Silk Road Investment Group Co. Ltd., Xi'an, China; ^4^Sino-Australia Joint Research Centre in BIM and Smart Construction, Shenzhen University, Shenzhen, China; ^5^Department of Civil Engineering, College of Engineering and Physical Sciences, Aston University, Birmingham, United Kingdom

**Keywords:** prefabrication, contractor, key factors, social network analysis, China

## Abstract

Adoption of prefabrication is essential for improving the urban built environment. However, the existing prefabrication market in China is far from mature. As the stakeholder who conducts construction activities, the contractor is facing a dilemma of lacking steady prefabricated components supply. In this circumstance, a potential solution is that contractors open their own prefabrication factories to guarantee stable component supply. The aim of this research is exploring the key factors for contractors to open prefabrication factories. Firstly, a total of 28 influencing factors were identified from literature. Then, the identified factors were divided into four categories: policy environment, market environment, technological environment, and enterprise internal environment. Through interviews with experienced professionals, a total of 19 factors were selected for future analysis. Based on the 19 factors, a questionnaire was designed and distributed to the experts to rate the degree of mutual influences. The collected data were analyzed using Ucinet6.0 software, and the adjacency matrix and the visual models were established. Finally, through the analysis of node centrality, betweenness centrality, and closeness centrality, the four key influencing factors were determined including mandatory implementation policy, precast concrete component's price, market demand, and contractor's strategic objectives. The results of this study could assist contractors in making decisions of opening their own prefabrication factories toward more sustainable environment.

## Introduction

A prefabricated building refers to a building in which all or parts of the building are prefabricated in a factory. These are then transported to construction sites for assembly, connection, and partial cast-*in-situ* construction (1–3). Compared with traditional construction methods, prefabrication has many advantages (4). First, using prefabricated components is a way to industrialize construction, which greatly improves the duration, quality, and sustainability of the project. It also reduces waste, noise, dust, operation cost, labor demand, and resource depletion of the construction site. In addition, the main components of the prefabricated building are produced in factories, while the assembly of components can be carried out at the same time with other *in-situ* construction processes (5). Bottlenecks and construction delays are common problems due to inefficient construction site production. Prefabricated components can reduce bottlenecks, improve production rates, and shorten production times (6). In addition, weather does not have significant negative impacts on prefabricated construction assembly and this effectively ensures construction efficiency (7). Furthermore, relevant research results show that prefabricated components generate savings ranging from 4 to 14% of total life-cycle energy consumption. The surrounding urban built environment also benefits from waste reduction and quality control (8). Prefabricated components can be used to control pollution since the pollution resulting from site work has become a big threat to the urban environment. Prefabricated components are constructed on-site by lifting and splicing and thus reduce the workload for *in-situ* construction, improve the quality control process, and ensure the health and safety of workers (9). Due to the high degree of standardization of prefabricated components, it is possible to adopt digital technologies and information management in the design, production, transportation, and assembly process.

Development of prefabricated construction in China has been ongoing for over 70 years (10–12). However, the development of prefabrication progressed at a slow pace due to some challenges such as quality problems, higher prefabricated component cost (13), and rapid development of *in-situ* construction (14). Prefabrication is linked in several ways to factory production, assembly construction, information management, and intelligent applications (15). In recent years, with a declining labor force, an increase in employment costs and national sustainable development requirements (16), prefabricated construction has gained more attention. It plays a critical role in realizing the industrialization and modernization of the construction industry (17). Thus, prefabrication has been widely implemented and promoted in China over the past few years.

To promote prefabrication development, the national government has put forward a development goal of having prefabricated buildings make up 30% of all newly built buildings in about 10 years (18). In response to this development goal, more than 30 cities including Shanghai, Chongqing, and Beijing have issued a series of policies to promote prefabrication. In addition, standards and norms have been introduced to promote the implementation of prefabricated construction. They have been shown to play an important role in safeguarding the development of prefabrication in China (19). According to officially released data, a total of 630 million square meters of prefabricated buildings were built in 2020, which accounted for 20.5% of all newly built buildings (20).

Along with the rapid promotion of prefabricated buildings, industries related to prefabrication have developed quickly as well. According to government statistics, a total of 328 national-level prefabricated construction industrial bases and 908 provincial-level industrial bases have been established across the country, which indicates that the market demand for prefabricated components is huge (21). To deal with such a huge demand, prefabrication factories have been opened by different stakeholders including developers, design companies, contractors, and other investors (22). As the stakeholder who is directly involved in the construction process, the contractor has the willingness to open prefabrication factories to preferentially fulfill demand (23). However, as opening a prefabrication factory requires a huge amount of resources (e.g., site, technology, investment), the contractors need to carefully consider their potential decisions of opening prefabrication factories from many aspects. In existing literature, there is a knowledge gap of identifying what are the key factors for contractors to make decisions. Thus, this paper aims to explore the key factors contractors take into consideration when deciding to open prefabrication factories.

## Literature Review

### Identification of Potential Factors

Potential influencing factors were extracted from previously published relevant literature. After an initial screening, a total of 28 potential influencing factors were identified. Five experienced professionals were then invited to filter the identified potential influencing factors and their opinions on these factors were solicited. Based on their suggestions and comments, nine potential influencing factors were excluded and the remaining 19 factors were classified into four categories according to their attributes as shown in Table 1.

**Table 1:** Summary of influencing factors.

**No**.	**Category**	**Influencing factor**
1	Policy environment	Mandatory implementation policy
2		Economic incentives for manufacturing
3		Availability of priority land supply
4	Market environment	Market demand
5		Industrial chain maturity
6		Competitive pressure
7		Developers' acceptance of prefabricated buildings
8		Price of precast concrete components
9		Industrial support
10	Technological environment	Contractor's professional competence
11		Maturity of standards
12	Enterprise internal environment	Scale of contractor enterprise
13		Strategic objectives of contractor
14		Perception of contractor
15		Innovative potential of contractor
16		Willingness of contractor
17		Precision manufacturing
18		Expected economic benefits for contractor
19		Quality of construction

### Explanation of the Influencing Factors

The influencing factors can be explained as follows.

#### Policy Environment

To develop industrialized construction in China, a mandatory implementation policy has been promulgated. According to the 2015 Construction Industry Modernization Development Outline, prefabricated buildings will account for more than 30% of new buildings by 2025 (24). The mandatory implementation policy was promulgated to upgrade existing industrial structures to satisfy green development goals. In essence, the aim of the mandatory implementation policy was to change the traditional construction mode to the prefabrication method, which is an important measure for promoting supply-side structural reform (25). In short, the policy is an essential driving force for promoting the application of prefabrication construction in the construction industry (26).

Economic incentives for manufacturing such as tax reductions have been continuously emphasized. Liang (27) stated that the total value of tax reduction was approximately 2.36 trillion yuan in 2019, which was essential to promote the high-quality development of the manufacturing industry. As equipment accounts for a huge proportion of total costs, a tax reduction policy could help to decrease the total cost and promote high-quality development.

The availability of priority land supply refers to giving priority to providing land for prefabrication factory sites. On the basis of the 2019 Industrial Land Policy Implementation Guidelines, the land supply policy incentive was provided to contractors for transformation and upgrading by local governments (28). Zhou et al. (29) stated that industrial land supply has political connotations as a critical tool in China.

#### Market Environment

Relevant research shows that opening prefabrication factories is aligned with current market demand in China. This is because the annual market size for the nationwide industrialization of construction is expected to reach 5 trillion yuan in 2025, which would account for about 50% of the total output value of the entire construction industry (30). Market demand refers to the need for prefabricated components which promote technological innovation. The next 10 years will be a golden period of rapid development in the industrialization of construction.

The level of industrial chain maturity directly affects the benefits of contractors, which is based on the relationship between market supply and demand. There are two key nodes in the industry chain, namely the prefabricated components in the production stage and the sales stage (31). Industrial chain maturity in this article refers to a new prefabricated industry chain which is a key part of the construction process.

The competitive pressure for contractors in the construction market is sustained growth, since the construction industry is subject to strong internal competition. The increasingly competitive environment has put various contractors under great pressure. Opening prefabrication factories is the right way to provide developers with better products and services. Contractors can also acquire core competitive prefabricated technology to outshine market competition (32).

To a significant extent, the demand for prefabricated buildings depends on the willingness of developers to accept them. They are the upstream enterprises supplying contractors in the industry chain (33). The willingness of developers to accept prefabricated buildings has led to prefabrication methods that have gone beyond traditional construction constraints. With increasing acceptance of prefabricated technology, contractors will have many new opportunities and challenges in the construction market.

The price of precast concrete components is a vital factor in the cost of any building. This refers to the cost management of prefabricated projects (34). Jiang et al. (35) stated that the selection of prefabricated component suppliers is one of the important links in the housing industry chain and the price of prefabricated components is one of the key factors. The choice on whether to open prefabrication factories or not depends on the profit earned from prefabrication sales.

Industrial support refers to the relevant industrial conditions for prefabricated production, such as the supply of raw materials, transportation conditions, and industrial workers (36). The completeness of industrial support conditions in the region affects the investment, construction, and production costs of prefabricated component factories. Insufficient industrial support capacity will lead to a lack of relevant enterprises in the regional industrial chain and increase the operating costs of enterprises.

#### Technological Environment

The professional competence of the contractor refers to its technical competence in the prefabricated construction field. It is critical to acquire various key construction techniques to keep up with cutting-edge technology, including systems, specialization, integration, prefabrication, assembly, and information technology (37). Opening prefabrication factories can help improve the professional level of contractors in the prefabricated construction field. Furthermore, the professional competence of a contractor includes its human resources department, as “having the right people” is crucial for success. The financial and quality outcome of a project is highly dependent on the competence of the individual chosen as the site manager (38).

It is crucial to make perfect standard specifications which are related to the design, production, and installation of prefabricated buildings. Vakili (39) stated that maturity of standards was conducive to the production, operation, and sales of prefabricated component factories. Moreover, standards establish requirements that stipulate prefabricated construction processes and products. Related design and technical standards can provide professional expertise to guide contractors in the implementation of prefabricated construction (40).

#### Enterprise Internal Environment

The internal environment of the company refers to the economic and technological level of the enterprise which may sway its decision to open prefabrication factories. There is no obvious gap in policy awareness among various sectors, so large companies do not have a better understanding of market-based instruments than their small and medium-sized counterparts (41). Li et al. (42) stated that firm size has a significant effect on the willingness of construction enterprises to accept the policy. Small enterprises are restricted by many factors such as capital, technology, and talent. The strategic goals of a contractor guide the direction of development of an enterprise.

Building prefabrication was strongly promoted by local governments, which relies on the *in-situ* manufacturing and work-site assembly of prefabricated components (17). The strategic objectives of contractors should be aligned with local policies, so that the green benefits of prefabricated building initiatives can be reaped in China. The establishment of prefabrication factories can expand the business scope of the enterprise and enhance the level of specialization in prefabricated buildings. This is conducive to the development of EPC (Engineering Procurement Construction) and other general contracting methods (43).

Prefabricated buildings have the potential for improved quality, productivity, efficiency, safety, and sustainability (44). The perception of contractors has been further enhanced under the mandatory implementation policies. Yu et al. (45) stated that a perception of greater purchasing behavior led to more actual purchasing behavior to a limited extent, which is analogous to the building behavior of contractors who work with prefabrication factories.

The innovative potential of a contractor refers to the innovation present in the construction technology or assembly technology used by the contractor. The level of innovation of Chinese construction enterprises is not ideal, and the innovative potential of construction enterprises must be urgently strengthened at this stage (46). Building prefabrication factories and promoting contractor transformation can effectively enhance the innovative potential of contractors (47).

According to the findings of field interviews, research and applications, prefabricated technologies are deeply affected by the market environment, especially company awareness of prefabrication buildings and prefabrication factories. The willingness of contractors to open prefabrication factories depends on market demand. The competitiveness of traditional buildings is still higher than that of prefabricated buildings in China (48). Huang et al. (49) stated that it was influenced by many factors. The more willing the contractors, the greater the possibility that they would establish prefabrication factories.

The production of prefabricated components is the link between design and construction. It is essential for prefabrication factories to achieve precision manufacturing and quality excellence. The realization of precision manufacturing can effectively avoid or even eliminate waste and uncertainty in the production process, which can improve product quality and production efficiency (26, 50). Contractors have technical and management advantages when establishing prefabrication factories that can help to realize in-plant precision manufacturing and lean construction assembly of prefabricated components at work sites.

Most contractors often pursue the economic benefits derived from developers because the ultimate goal of contractors is to earn a profit. The cost of prefabricated construction is higher than traditional construction due to the uneven distribution of prefabrication factories and high operation costs in China (51). Prefabrication and industrialized building systems confer advantages including shorter project duration, cost savings, enhanced site protection, better product quality, and reduced waste (52). For contractors, opening prefabrication factories can enhance their own industrial capacity. This proposal takes into consideration the life cycle cost and the increased benefits of the final product.

The aim of promoting the sustainable development of prefabricated buildings is to satisfy current and future needs of the construction industry. It also ensures faster progress, cost-effectiveness and construction quality, as well as worker safety (53). Therefore, contractors will pay more attention to construction quality as producing standardized and high-precision prefabricated components can effectively avoid errors caused by manual labor in traditional construction. Furthermore, prefabricated construction technology can effectively optimize construction structure and improve construction quality (54, 55).

## Research Methodology

### Data Collection

In this study, 11 experienced experts from different stakeholders, such as government (1), research institutes (2), contractors (4), and prefabrication factories (4), were invited to gives scores for the degree of influence of various factors by completing questionnaires. All of the invited experts must have professional experiences more than 5 years and present different stakeholders' viewpoints on opening a prefabrication factory. The first row and the first column of the matrix are the influencing factors, and the values in the matrix represent the degree of influence that each element in the columns has on the elements in the rows. In the questionnaire, the experts were asked to give a score ranging from 0 to 4 to evaluate the degree of influence. A score of 0 means that the element in the column does not influence the element in the row; a score of 1 means the degree of influence is small; a score of 2 means the degree of influence is average; a score of 3 means the degree of influence is large; and a score of 4 means a very high influence.

After collecting the questionnaire results from the experts, Ucinet 6.0 software was used to conduct a consistency analysis to check the expert scoring results. Ucinet 6.0 software was selected because it can deal with the original data as the format of matrix and then provide visible relationships between different influencing factors.

### Social Network Analysis

According to the social network theory, actors are resource-competitive and form social relations and social network structures through resource flows. Therefore, the characteristics of the network structure and relationships in the network have important impacts on the actors (56). The relationship structure of social networks can be generally represented by graphs or matrices. There are three main methods of representation used in this article, namely degree centrality, betweenness centrality, and closeness centrality. Among them, degree centrality refers to the degree to which a node is directly related to other nodes. The greater the number of nodes, the greater the power of the node in the social network. The betweenness centrality refers to the ability of a node to control other nodes. The greater the centrality, the larger the number of nodes that need to pass information between each other via this node. The closeness centrality is the reciprocal of the sum of the distances from the node to all other nodes. The larger the value, the easier it is for the node to communicate with other nodes to transfer resources. Social network analysis solves the complex interest relationships between actors or organizations in real life by quantitatively analyzing the social relationships between actors. Therefore, it is widely used in various research fields, such as economics (57), sociology (58), and management (59).

In the field of construction engineering, the decision-making of contractors is influenced by many actors, such as governments, developers, and component manufacturers. This study used social network analysis to identify the power and reputation of actors in the decision-making mechanism for contractors. It provided a basis for the selection of the main body of the social network organization structure. Meanwhile, it can effectively identify key influencing factors in the behavioral decision-making mechanism, analyze the degree of mutual influence, and analyze the decision-making mechanism for contractors when deciding to open prefabrication factories (60).

#### Network Density Analysis

Network density can reflect the degree of connection between nodes in a network. If the overall social network graph is an undirected relational network with n influencing factors, the theoretical value of the total number of associations is *N* ^*^ (*n* – 1)/2. If the actual number of associations (which can be considered as the number of connections) contained in the social network is *M*, the social network density is then equal to *m*/(n^*^(*n* – 1)/2) = 2*m*/(*n*/(*n* – 1)). If the whole social network graph is a directed relational network, the theoretical value of the total number of associations is *N* ^*^ (*n* – 1) and the network density is equal to *M*/(*n*/(*n* – 1)) (61). In particular, the formula for network density in the directed relational network is:


(1) $$\begin{array}{r} Den=\frac{m}{n\left(n-1\right)} \end{array}$$

#### Degree Centrality Analysis

In the social network diagram, degree centrality refers to the number and intensity of direct or indirect connections (adjacent connections) with a certain node. If the node is located in the center of the social network diagram, the value for degree centrality of this node increases, and more nodes are connected with that point. Degree centrality aims at finding centrality based on the notion that important nodes have many connections as expressed in Equation (2) (61).


(2) $$\begin{array}{r} C_{D}\left(p_{k}\right)=\sum_{i=1}^{n}a\left(p_{i},p_{k}\right) \end{array}$$

where *n* is the number of nodes in the network and *a*(*p*_*i*_, *p*_*k*_) is a distance function. *a*(*p*_*i*_*, p*_*k*_) = 1 if and only if node *p*_*i*_ and node *p*_*k*_ are connected. Otherwise, *a*(*p*_*i*_, *p*_*k*_) = 0.

#### Betweenness Centrality Analysis

Betweenness centrality aims at finding centrality based on the assumption that nodes that connect other nodes are important nodes. Betweenness centrality measures the ability of an influencing factor to control other factors, i.e., other factors can only be related through this factor, and nodes with high betweenness centrality are in the center of the network. The larger the shape of the node, the greater the betweenness centrality of the influencing factor, i.e., the closer to the center of the network the influencing factors are, the stronger the degree of influence on other nodes. The formula used to calculate the degree of centrality is shown in Equation (3), which is used to express betweenness centrality (61).


(3) $$\begin{array}{r} C_{B}\left(p_{k}\right)=\sum_{i<}^{n}\sum_{j}^{n}\frac{{\text{g}}_{\text{ij}}\left(P_{k}\right)}{{\text{g}}_{\text{ij}}},i\neq j\neq k \end{array}$$

where *g*_*ij*_ is the number of geodesics (shortest paths) linking node *p*_*i*_ and node *p*_*j*_ and *g*_*ij*_(*p*_*k*_) is the number of geodesics linking node *p*_*i*_ and node *p*_*j*_ that contains node *p*_*k*_ (62).

#### Closeness Centrality Analysis

The closeness centrality refers to the ability where the influencing factor cannot be controlled by other factors. It is derived by calculating the sum of the shortest distance between a certain node and all other nodes in the entire network. When the distance between a node and other nodes in the network model is short, it proves that the node is highly capable of mastering and conveying information instead of being easily affected by other nodes. The value can be normalized by using the maximum possible distance between any two nodes in a network of *n* nodes. This value is *n* – 1. More precisely, the normalized closeness of node *p*_*k*_ is given by (61):


(4) $$\begin{array}{r} C_{c}\left(p_{k}\right)=\frac{{\sum_{i=1}^{n}d\left(p_{i},p_{dk}\right)}^{-1}}{n-1} \end{array}$$

## Results and Discussion

Based on the results of the consistency analysis, this study optimizes the scoring data of the experts and obtains a social network analysis matrix (adjacent matrix) of the factors affecting the decision-making mechanism of contractors when it comes to opening a prefabrication factory.

### Network Density Analysis Results

**Figure 1:**
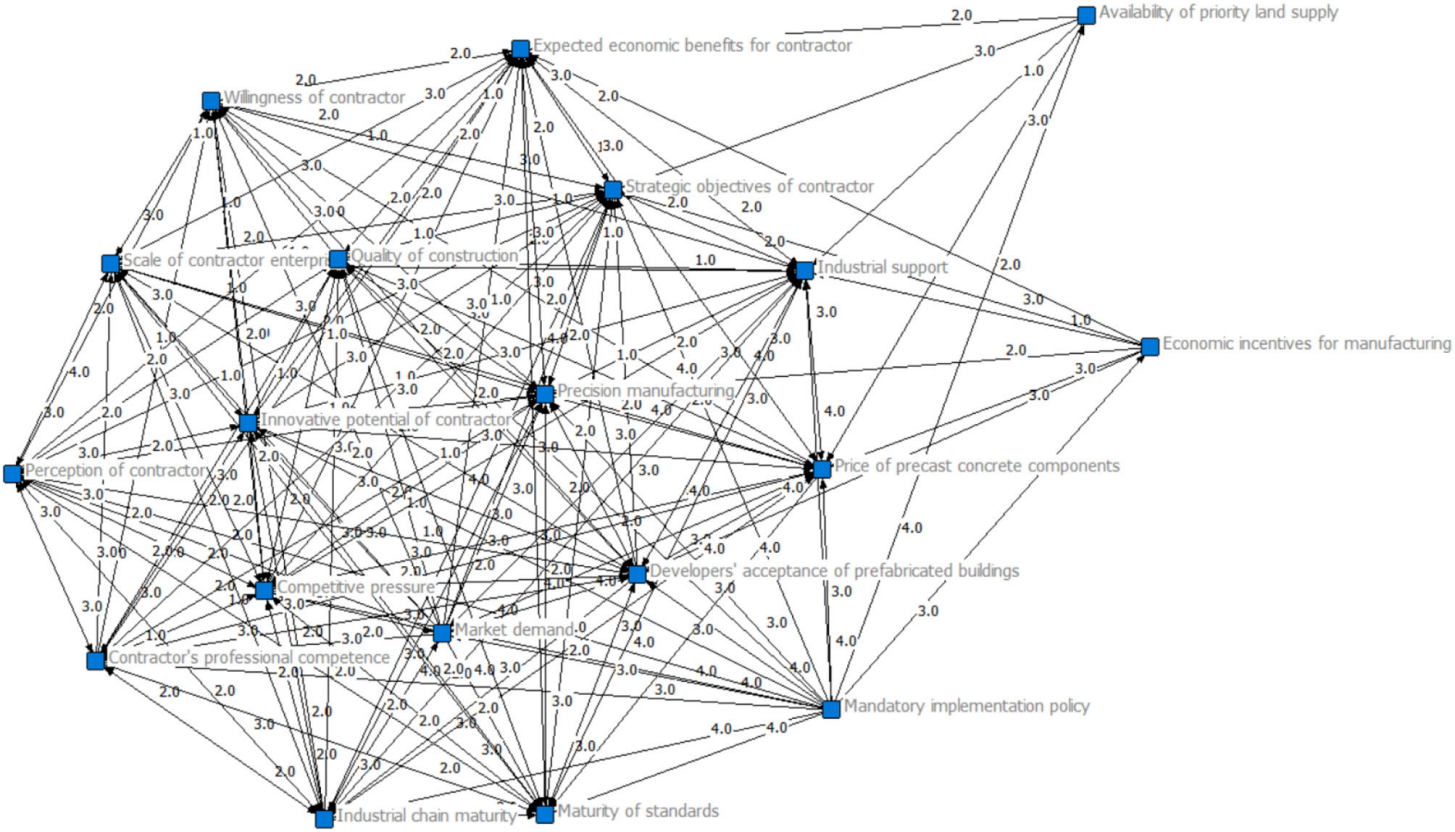
Visual model of influencing factors in a relationship network.

Based on the adjacency matrix of influencing factors, Ucinet 6.0 was used to visualize the network association of influencing factors as shown in Figure 1. In Figure 1, there are directional correlations between the influencing factors. The relationships among the influencing factors are indicated by arrows and the number on a specific arrow represents the degree of influence for each node (63).

According to the formula, the network density of the model is 1.4620, indicating that the network graph of influencing factors for the decision-making mechanism regarding the opening of fabrication factories by contractors has a high density (generally, 0.50 is used as the average standard for measuring density). In other words, the network graph of influencing factors changes as a whole with a change in any two factors. Therefore, through the analysis of network density, it can be found that the change in the relationship network caused by the change of each influencing factor. We can then modify the decision-making mechanism for contractors when deciding whether to open a prefabrication factory.

### Degree Centrality Analysis Results

**Figure 2:**
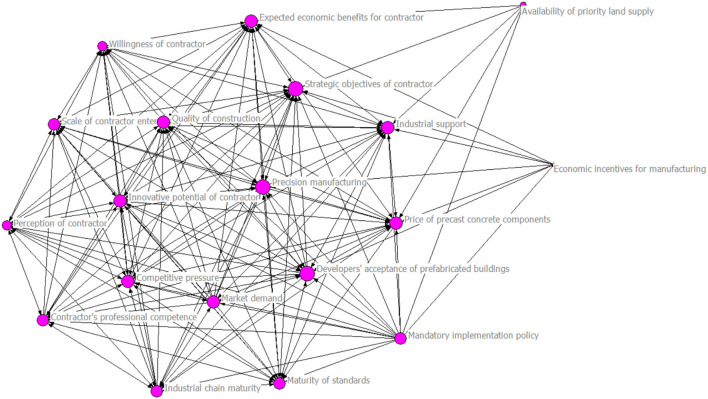
Visual model for influencing factors that affect the degree centrality result.

The visualization result for degree centrality analysis is shown in Figure 2. The larger the node in Figure 2, the higher the degree centrality of the node. Degree centrality is measured by node in-degree and node out-degree. Node in-degree indicates the number and correlation of other influencing factors directly affecting this factor, and node out-degree indicates the number and correlation of other factors that are directly affected by this influencing factor.

Based on the degree centrality analysis results of the influencing factors, the 19 influencing factors are sorted in a descending order based on their level of out-degree as shown in Table 2. They can be divided into four categories according to their levels of out-degree and in-degree:

a) The out-degree of an influencing factor is large and its in-degree is small. This means that the influencing factor can easily affect other factors, but is not easily affected by other factors (64). It is a spontaneous influencing factor and is the source of influence in the relationship network.
b) The out-degree of an influencing factor is small and its in-degree is large. This means that the influencing factor does not easily affect other factors, but is easily affected by other factors.
c) The out-degree of an influencing factor is large and its in-degree is large. This means that the influencing factor easily affects other factors and is also easily affected by other factors.
d) The out-degree of an influencing factor is small and its in-degree is small, this means that the influencing factor neither easily affects other factors, nor is it easily affected by other factors.

**Table 2:** Analysis results of degree centrality for influencing factors.

**Sorting**	**Influencing factor**	**Out-degree**	**In-degree**
Out-degree is large and in-degree is small	Mandatory implementation policy	54.00	0.00
	Market demand	44.00	17.00
	Perception of contractor	30.000	26.000
	Professional competence of contractor	29.000	20.000
Out-degree is large and in-degree is small	Developers' acceptance of prefabricated buildings	36.000	34.000
	Price of precast concrete components	32.000	42.000
	Industrial chain maturity	31.000	30.000
	Competitive pressure	29.000	34.000
	Maturity of standards	28.000	34.000
Out-degree is small and in-degree is large	Strategic objectives of contractor	25.000	44.000
	Expected economic benefits for contractor	23.000	34.000
	Precision manufacturing	23.000	32.000
	Scale of contractor enterprise	22.000	31.000
	Industrial support	21.000	30.000
	Willingness of contractor	11.000	36.000
Out-degree is small and in-degree is small	Innovative potential of contractor	20.000	24.000
	Quality of construction	19.000	25.000
	Economic incentives for manufacturing	14.000	3.000
	Availability of priority land supply	9.000	4.000

The out-degree and in-degree of the influencing factors are relatively small. This means that such factors do not easily affect other factors and are not easily affected by other factors, meaning that they are independent control factors. Such factors include the innovative potential of a contractor, construction quality, availability of priority land supply, and economic incentives for manufacturing. To improve the role of these factors, it is necessary to start from the factors themselves, such as promoting the development of innovation capabilities and improving innovation through incentives such as efficiency measures.

#### Influencing Factors With a Higher Out-degree and Lower In-degree

According to the research findings, the influencing factors with higher out-degree and lower in-degree include mandatory implementation policy, market demand, perception of contractors, and professional competence of contractors. Among them, the mandatory implementation policy has the largest out-degree and the smallest in-degree, indicating that this factor is only subject to government management and regulation as an external influence. It can have an impact on most of the factors related to the decision-making mechanism of contractors when opening prefabrication factories. Generally, market demand is only affected by policy environment factors, which will affect the decision-making of the contractor when it comes to factory construction. The perception of the contractor is generally affected by the mandatory policy environment, so its in-degree is relatively small. However, it will have a limited impact on the internal environmental factors of the contractor enterprise, such as the strategic goals of the contractor and the willingness of the contractor to build a factory, so the out-degree is relatively large. The professional competence of the contractor is an attribute of the contractor and is generally only affected by the internal environment of the enterprise, but it will have an impact on the developer and the market environment. For example, the stronger the level of technical competence when it comes to prefabricated construction for the contractor, the more it can promote the acceptance by developers of prefabricated buildings and market recognition of prefabricated buildings. Many cities in China are facing challenges associated with low-carbon transformation (65). As a significant contributor of carbon emissions, the construction industry has been subject to a series of policies promulgated by local governments which encourage them to be more industrialized (66).

#### Influencing Factors With a Lower Out-degree and Higher In-degree

According to the research findings, the factors associated with small out-degree and large in-degree include the strategic objectives of the contractor, expected economic benefits for the contractor, precision manufacturing, size of contractor enterprise, industrial support, and willingness of the contractor. Such influencing factors do not typically affect other influencing factors, but they are easily affected by other factors. For contractors, their strategic objectives determine their development route, but this is easily affected by changes in mandatory policy and market demand. On the other hand, market demand and mandatory policy do not change with the strategic objectives of the contractor. One of the purposes behind the willingness of a contractor to construct a factory is to reduce the cost of the prefabricated project and obtain more economic benefits. The economic benefits are also affected by the market, technology and policy environment. Some internal environmental factors such as the strategic objectives of the contractor, the expected economic benefits of the contractor, precision manufacturing, size of the contractor enterprise, and the willingness of the contractor to build a factory are easily influenced by policy and market factors. However, they are unlikely to change policy and market factors. Industrial support refers to the upstream enterprises related to the prefabricated component industry within the region. The strength of industrial support is affected by many factors, such as raw material supply conditions of prefabricated components, transportation conditions of prefabricated components, and surrounding production environment conditions. Policy and market factors directly affect related industrial support and the expected economic benefits of contractors after building factories. Precision manufacturing of the contractors is affected by the size of the contractor enterprise and professional competence of the contractor.

#### Influencing Factors With a Higher Out-degree and Higher In-degree

According to the research findings, the factors associated with large out-degree and large in-degree include developer acceptance of prefabricated buildings, price of the precast concrete components, industrial chain maturity, competitive pressure, and maturity of standards. Developer acceptance of prefabricated buildings directly affects the behavioral decisions of contractors in deciding whether to open fabrication factories. It is also affected by mandatory implementation policy, market demand, and the professional capabilities of contractors. For the contractor, the establishment of prefabrication factories is conducive to improving industrial chain maturity, so as to meet market demand and increase its market competitiveness. It also conforms to national policy guidance for the promotion of prefabricated construction. Developer acceptance of prefabricated buildings not only affects the size of the contractor enterprise and strategic objectives of the contractor, but is also affected by policy and market environment factors. Competitive pressure mainly affects the enterprise's internal environment, such as the strategic objectives of the contractor and the innovative potential of the contractor. It is also affected by the policy environment and the market environment. The price of precast concrete components affects the expected economic benefits of the contractor and the willingness of the contractor to open prefabricated factories. It is closely related to the market and policy environment. With the development of prefabricated buildings, relevant standards, and specifications are constantly being improved. With standards and specifications, a market environment and technological environment can be developed. The formulation of the corresponding rules is also beneficial to contractors when establishing prefabrication factories.

#### Influencing Factors With a Lower Out-degree and Lower In-degree

According to the research findings, the factors associated with small out-degree and small in-degree include innovative potential of the contractor, construction quality, availability of priority land supply, and economic incentives for manufacturing. To improve the role of these factors, it is necessary to start from the factors themselves, such as promoting the development of contractor innovation capabilities and improving innovation and efficiency incentives for contractors. It is also important to strengthen the implementation of preferential land supply and economic incentive policies for the manufacturing industry. Honorary incentive policies for quality construction of high-quality projects should also be encouraged.

### Betweenness Centrality Analysis Results

**Figure 3:**
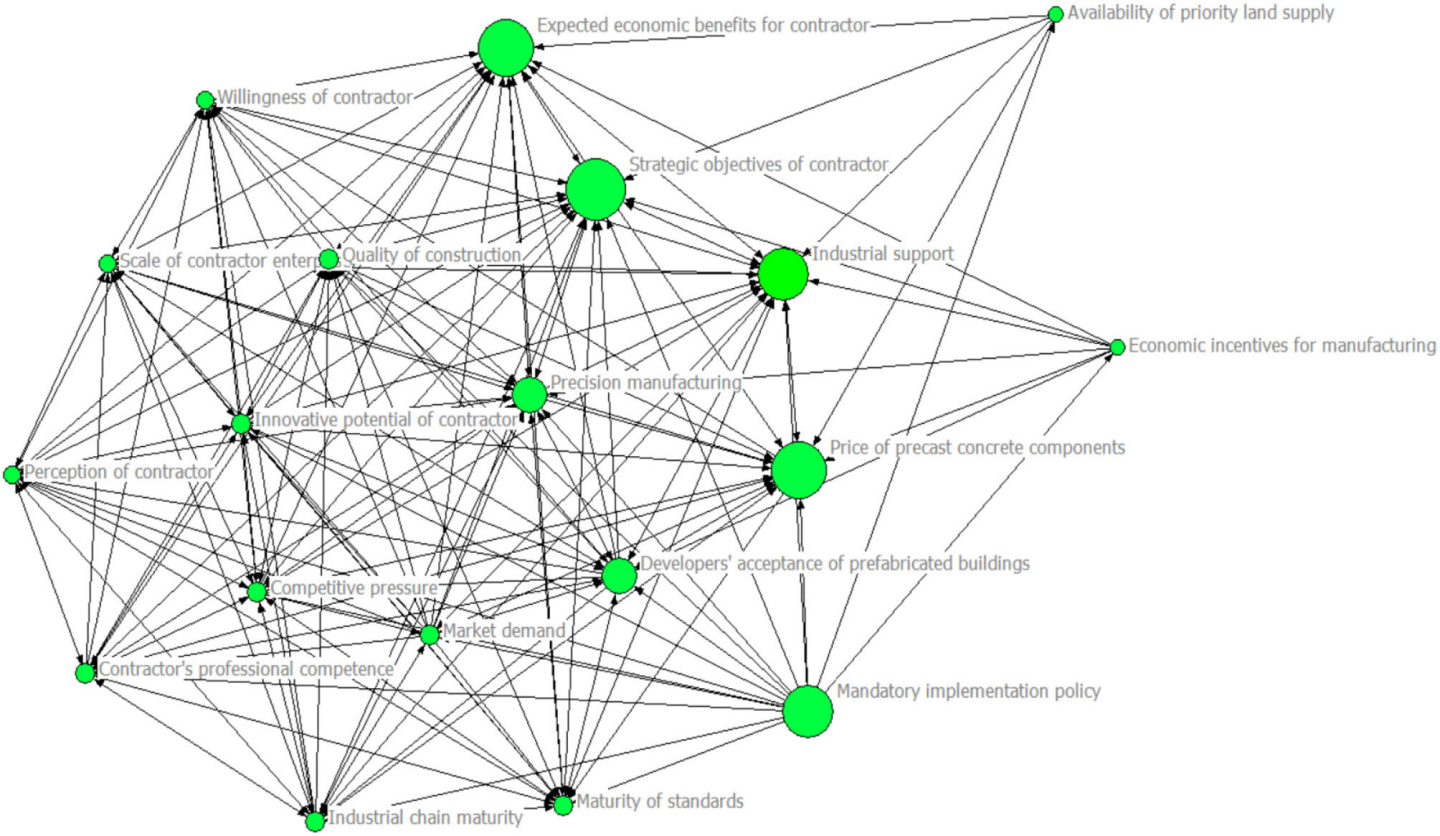
Visual model of betweenness centrality results for influencing factors.

The visualization results of the centrality analysis of influencing factors are shown in Figure 3. Based on the analysis of the betweenness centrality of the influencing factors, the 19 influencing factors are sorted according to their level of betweenness centrality as shown in Table 3. The average value of the betweenness centrality is 4.789. Overall, the betweenness centrality scores of the influencing factors are highly polarized. Among them, the strategic objectives of contractors, industrial support, developer acceptance of prefabricated buildings, expected economic benefits of contractors, and competitive pressure are higher than average. This indicates that these factors have strong control capabilities and serve as a bridge for other influencing factors to generate multiple correlations.

**Table 3:** Analysis results of betweenness centrality for influencing factors.

**Order**	**Influencing factor**	**Betweenness**
1	Strategic objectives of contractor	12.507
2	Industrial support	11.325
3	Developers' acceptance of prefabricated buildings	10.166
4	Expected economic benefits for contractor	9.479
5	Competitive pressure	7.608
6	Scale of contractor enterprise	5.739
7	Price of precast concrete components	5.726
8	Precision manufacturing	5.593
9	Industrial chain maturity	5.559
10	Maturity of standards	4.593
11	Professional competence of contractor	3.429
12	Quality of construction	2.39
13	Innovative potential of contractor	1.946
14	Market demand	1.94
15	Perception of contractor	1.574
16	Willingness of contractor	1.272
17	Economic incentives for manufacturing	0.077
18	Availability of priority land supply	0.077
19	Mandatory implementation policy	0
Average		4.789

The betweenness centrality of maturity of standards, professional ability of contractors, construction quality, contractor innovation ability, market demand, contractor awareness of building factories, contractor willingness, manufacturing economic incentives, availability of priority land supply, and mandatory implementation policies are lower than the average value. This indicates that the conduction effect of these factors in the network relationship is weak. Among them, the betweenness centrality of policy factors is 0 or almost 0, which means that almost no relationship between any two influencing factors is transmitted through the influencing factors, so they are at the edge of the network and are hardly connected to other factors.

### Closeness Centrality Analysis Results

**Figure 4:**
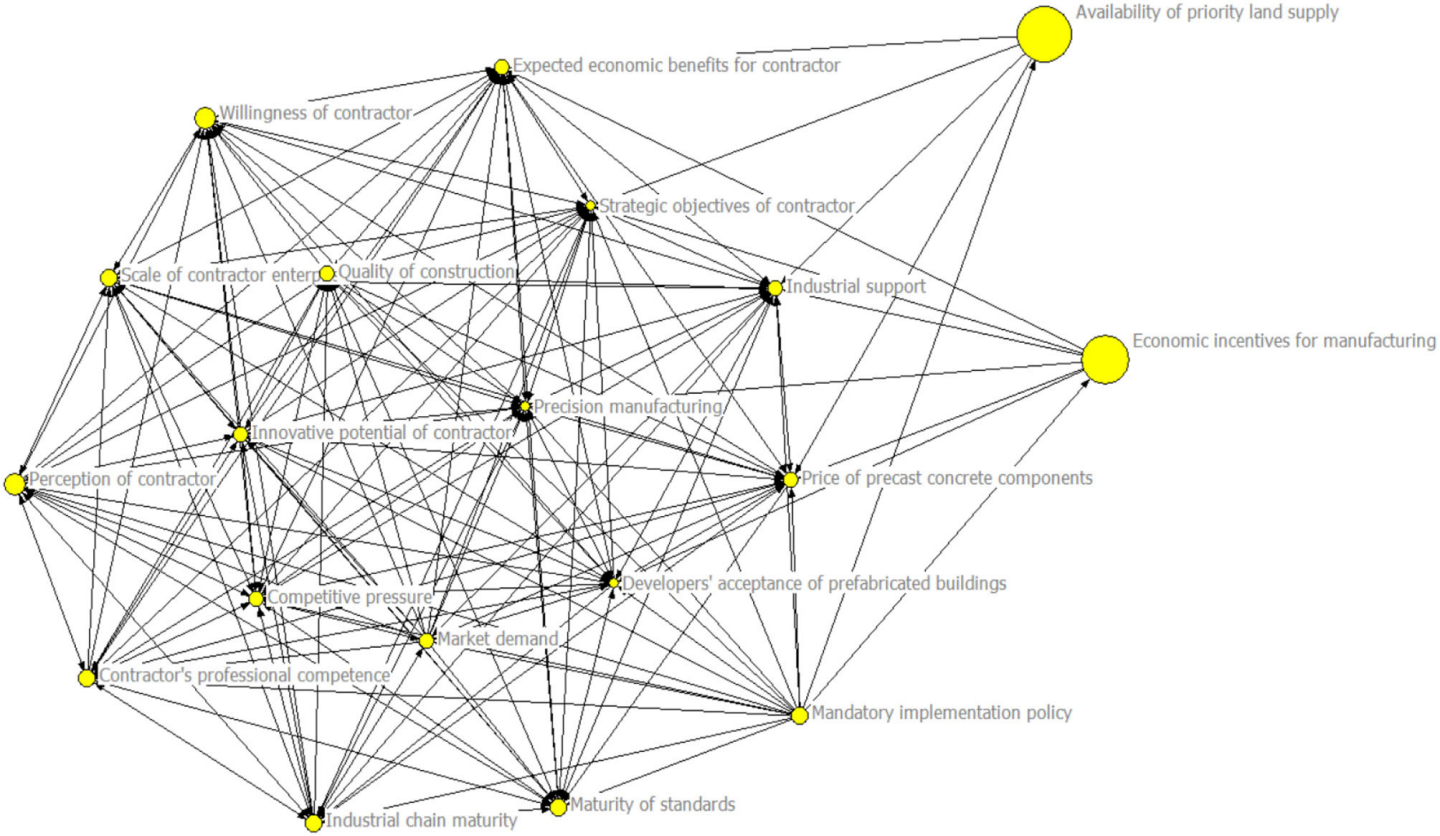
Visual model of closeness centrality results for influencing factors.

The visualized results of the closeness centrality for influencing factors are shown in Figure 4.

According to Table 4, the closeness centrality of the influencing factors can be divided based on two indicators: in-closeness centrality and out-closeness centrality. In-closeness centrality means how easy it is for other nodes to reach the node, while out-closeness centrality refers to how easy it is for the node to reach other nodes. These two representations are the reciprocal of the sum of the shortest distance. In general, low in-closeness centrality and high out-closeness centrality indicate that it is not easy for other nodes to reach this node. However, it is easier for this node to reach other nodes. Therefore, the independence of resource output will be high at the edge of the network if the influencing factors have low in-closeness centrality and high out-closeness centrality. High in-closeness centrality and low out-closeness centrality mean that it is easier for the other node to reach this node. It is more difficult for this node to reach other nodes. Therefore, influencing factors with high in-closeness centrality and low out-closeness centrality mainly depend on the resource input of other subjects in the network in the central part of the network.

**Table 4:** Analysis results of closeness centrality for influencing factors.

**Influencing factor**	**In-closeness**	**Out-closeness**
Strategic objectives of contractor	94.737	23.684
Industrial support	85.714	23.077
Expected economic benefits for contractor	85.714	23.377
Precision manufacturing	85.714	23.684
Scale of contractor enterprise	81.818	23.684
Price of precast concrete components	81.818	22.785
Competitive pressure	81.818	24
Willingness of contractor	78.261	22.222
Quality of construction	78.261	23.077
Developers' acceptance of prefabricated buildings	75	24.324
Maturity of standards	72	23.684
Innovative potential of contractor	69.231	23.684
Industrial chain maturity	69.231	24.324
Professional competence of contractor	69.231	24.324
Perception of contractor	66.667	23.684
Market demand	58.065	25
Economic incentives for manufacturing	5.556	28.125
Availability of priority land supply	5.556	27.273
Mandatory implementation policy	5.263	85.714

Based on the analysis results of influencing factors regarding closeness centrality, the in-closeness centrality and out-closeness centrality with 19 influencing factors are summarized in Table 4. For mandatory implementation policies, the conclusion is that in-closeness centrality is low and out-closeness centrality is high. This shows that this influencing factor has high independence from the output of resources at the edge of networks. The strategic objectives of the contractor, industrial support facilities, expected economic benefits for the contractor, precision manufacturing, size of contractor enterprise, the price of precast components, and competitive pressure have relatively high in-closeness centrality and low out-closeness centrality. This means that they mainly depend on the input of resources from other subjects in the center of the network. There are 11 factors including contractor willingness to build prefabrication factories, construction quality, contractor acceptance of prefabricated buildings, maturity of standards, contractor innovation ability, industrial chain perfection, contractor professional competence, contractor perception of prefabrication mode, market demand, availability of land supply, and economic incentive measures that have relatively low in-closeness centrality and out-closeness centrality. It has been demonstrated that these influencing factors are relatively independent in the transmission of resources and are not easily controlled by other influencing factors and therefore they are at the edge of the network.

## Discussion

Based on the analysis results, four key influencing factors can be obtained through a comprehensive analysis of node centrality, betweenness centrality, and closeness centrality. More details are explained below.

It can be seen from the results that the mandatory implementation policy has the largest out-degree node as well as larger out-closeness centrality, which shows that this influencing factor tends to influence other factors instead of being easily controlled by other factors. Furthermore, it has a strong ability to dominate other influencing factors. Therefore, mandatory implementation policy can be considered as a key influencing factor in social networks as well as a source which is related to general influencing capability in the network. When node in-degree for mandatory implementation policy is 0, it means that the factor is not influenced by other factors. Mandatory implementation policies can only be decided by functional departments within national and governmental organizations. For example, in 2017, “Suggestions for the implementation on vigorously developing prefabricated buildings” aims to focus on promoting prefabricated buildings and developing new methods of construction.

The price of precast concrete components has the largest node centrality and large in-closeness centrality, which indicates that there are many factors closely connected with this factor. It takes the shortest distance for other influencing factors to reach this influencing factor in the social network. Therefore, the price of precast concrete is a key influencing factor in a social network. Meanwhile, price changes in precast concrete components will have a direct impact on capacity as well as market demands of prefabricated components companies. When starting construction, companies will consider the market price of precast concrete components and forecast expected economic benefits after the construction as references for planning construction project sizes. Moreover, the price of precast concrete components is also greatly correlated with transportation costs (67). Relevant research has shown that transportation costs in the suburbs are higher than those in urban areas (68–70). Therefore, the location where prefabricated components are constructed has a direct link with the economic benefits after construction.

Market demand has large betweenness centrality with low node centrality and closeness centrality. Therefore, it easily influences other factors and also has a strong ability to control other influencing factors instead of being easily influenced by them. This makes it a key influencing factor relating to networks. In the context of mandatory implementation policies, developers have started to develop more prefabricated building projects which lead to an increase in demand for prefabricated components. Construction market demand led by developers can have a positive and direct impact on opening prefabrication factories for contractors. The higher the developer acceptance of prefabricated buildings, the greater the demand for prefabricated components in the market. With the pursuit of economic benefits (63) as the aim, contractors will manufacture more prefabricated components based on market demand so as to meet the needs of developers. Market demand also has a positive impact on industrial chains. Relevant studies have demonstrated that if a company functions in a complex and uncertain business environment, the market demand of suppliers will have a direct and positive impact on the company industrial chains and enhance company performance (71, 72). For contractors, establishing factories for prefabricated components refers to a key link in the improvement of their own industrial chains for prefabricated buildings. After completing the factories, two important points which refer to both production and sales will be established in the industrial chains for prefabricated buildings, thus contributing to the development of companies. The method of lean management by contractors can be applied to production as well as sales to help effectively promote the development of enterprises.

Contractor strategic objectives have maximum betweenness centrality as well as in-closeness centrality with higher node centrality at the same time. This indicates that the factor tends to be influenced by other factors and easily influences other factors as well, thus having a “mesomeric effect” several times and becoming the “bridges” in social networks. Therefore, it is a key influencing factor in social networks. In the context of advancing economic globalization, the selection of development strategies becomes difficult for many companies. However, diversification and specialization are the main business strategies at present. When it comes to development routes of prefabricated buildings for contractors, establishing factories for prefabricated parts, expanding the business scope for companies, and improving specialization in prefabricated buildings will be helpful. In doing so, contractors can progress along the development path for projects involving general contracting as well as EPC which can integrate R&D, design, manufacturing, purchasing, and construction.

## Conclusions

Prefabricated construction has many advantages compared with traditional construction methods. However, the contractor may encounter difficulties in obtaining a steady supply of prefabricated components. Establishing self-owned prefabrication factories is a potential solution to solve this problem. The aim of this paper is to explore the key factors for contractors to open prefabrication factories. Relevant data were collected from questionnaires and further analyzed using Ucinet 6.0 to obtain the adjacency matrix and visual models of influencing factors. By using the social network analysis method, degree centrality analysis, betweenness centrality analysis, and closeness centrality analysis were carried out on the influencing factors. The analysis results revealed that mandatory implementation policy, price of precast concrete components, market demand, and contractor strategic objectives were the key factors that influence establishment of prefabrication factories by contractors. The results of this study contribute in revealing the potential mechanism for contractors to open prefabrication factories, thus to reduce carbon emissions and to promote sustainable development. However, this study also has a few limitations. For example, the research data were collected only from Guangdong province. Future research can be carried out across the country.

## Data Availability Statement

The original contributions presented in the study are included in the article/supplementary material, further inquiries can be directed to the corresponding author/s.

## Author Contributions

PX and JiasZ: conceptualization. JiaZ: methodology. JianZ: validation. PX, ZW, MA-A, and JiasZ: writing, reviewing, and editing—original preparation. All authors have read and agreed to the published version of the manuscript.

## Conflict of Interest

JiasZ and JiaZ are employed by China Construction Fourth Engineering Bureau No. 3 Co. Ltd., and JianZ is employed by China State Construction Silk Road Investment Group Co. Ltd. The remaining authors declare that the research was conducted in the absence of any commercial or financial relationships that could be construed as a potential conflict of interest.

## Publisher's Note

All claims expressed in this article are solely those of the authors and do not necessarily represent those of their affiliated organizations, or those of the publisher, the editors and the reviewers. Any product that may be evaluated in this article, or claim that may be made by its manufacturer, is not guaranteed or endorsed by the publisher.
